# Obesity-related microbial dysbiosis as a potential modulator of tumour progression

**DOI:** 10.1099/acmi.0.001055.v4

**Published:** 2025-10-21

**Authors:** Omar Mokhashi, Jaideep Chakladar, Wei Tse Li, Michael Karin, Matthew Uzelac, Weg M. Ongkeko

**Affiliations:** 1 Department of Otolaryngology-Head and Neck, University of California, San Diego, La Jolla, CA 92093, USA; 2 Research Service, VA San Diego Healthcare System, San Diego, CA 92161, USA; 3 UCLA School of Medicine, University of California, Los Angeles, CA 90095, USA; 4 UCSF School of Medicine, University of California, San Francisco, CA 94143, USA; 5 Laboratory of Gene Regulation and Signal Transduction, Department of Pharmacology, School of Medicine, University of California San Diego, La Jolla, CA, USA; 6 Stanford School of Medicine, Stanford University, Stanford, CA 94305, USA

**Keywords:** cancer, inflammasome, inflammation, microbiome, obesity

## Abstract

Although there is a well-established connection between the gut microbiome and obesity, the specific mechanisms by which microbes regulate cell signalling, inflammation and adipocyte growth to influence disease severity in obese patients remain largely unclear. Subsequently, while obesity itself is a well-established risk factor for various cancers, the exact mechanisms by which it drives disease progression are not yet definitively known. In this study, we explored the link between obesity-associated microbiome alterations and cancer progression by analysing microbial abundance in tissue samples from obese and cancer patients, and we identified specific microbes correlated with body mass index (BMI) that are associated with key cancer-related pathways. Notably, BMI-associated microbial species such as *Pseudomonas fluorescens* and *Lactobacillus sakei* were linked with both pro-tumour and anti-tumour progression in cancer patients. Additionally, microbes found to be abundant in cancer and obese tissue, such as *Pseudomonas baetica*, were significantly associated with the upregulation of certain oncogenic signalling pathways. BMI-associated microbes were also correlated with chemokine signalling and TFR2/NFkB-related genes. Both of these have well-established roles in inflammatory activity and inflammasome expression, a critical step in obesity-related cancer progression. Therefore, these microbes were found to be associated with variations in disease prognosis and patient survival. This study provides new insights into how obesity-related microbiome dysbiosis may be associated with cancer development and aims to introduce novel potential avenues for precision medicine approaches in cancer treatment.

## Data Summary

All RNA-sequencing data and mRNA expression count data for tumour tissue samples can be found in the GDC Data Portal (https://portal.gdc.cancer.gov/).

Clinical patient data can be sourced from the Broad GDAC Firehose (https://gdac.broadinstitute.org/), and genomic alteration details for each patient can be gathered from the Broad Institute's TCGA Genome Data Analysis Center report (http://gdac.broadinstitute.org/runs/analyses__latest/reports/).

Table S1 contains a comprehensive list of known immune-associated genes that were analysed in differential expression analysis.

## Introduction

Obesity is currently one of the biggest public health concerns in the USA and across the world, with a rapidly increasing prevalence [1]. In addition to the various symptoms it is associated with, it is also an established significant risk factor for a range of other chronic diseases, including various forms of cancer. In fact, this link in particular has garnered a lot of attention, largely due to the inflammation caused by obesity, which has been shown to help drive the development/progression of certain cancers [2,5]. However, despite this establishment and various research done on the topic, the specific underlying biological mechanisms connecting these two conditions are still not fully elucidated.

Prior research has shown how the human microbiome, a complex community of micro-organisms, primarily located in the gut, can directly influence body fat composition. A landmark study found that the transplant of intestinal microbes from mice raised in normal conditions to mice raised in a germ-free environment increased mice body fat content by 60% [6]. Further studies in mice reported that disruptions to the gut microbiota can promote or inhibit weight gain and that germ-free mice are more protected against diet-induced weight gains [7,8]. Verifying these findings in humans indicated a similar possible correlation of the microbiome to body fat composition, as *Bacteroides* and *Methanobrevibacter smithii* levels were found to be significantly reduced in obese human subjects in a 2012 study conducted on a cohort of obese patients in Marseilles, France [9]. This study, as well as subsequent ones, has established that dysbiosis, or the imbalance of microbial populations, is common in obesity and is also thought to contribute to the chronic low-grade inflammation characteristic of this condition, an established link between it and cancer [10,12].

Despite this, as well as several recent discoveries in these areas, the exact ways in which the microbiome influences body fat composition remain unclear, and our understanding of the microbiome’s role in obesity-related diseases is still in its early stages [13]. Moreover, the intra-tumour microbiome, or the abundance of distinct microbial species in tumour tissues, has been unexplored in cancer patients who are also obese. As such, profiling the intra-tumour bacterial population in cancer patients may provide critical insights into how microbes in cancer patients with obesity may lead to more severe disease. Recent reviews published by Yende and Sharma and Singh *et al*. have highlighted the influence of obesity-associated dysbiosis on cancer progression and immunotherapy efficacy in breast and colorectal cancer [14,15]. However, these studies are limited to single tumour types and do not directly correlate microbial species with host gene expression or distinguish between metabolically distinct obesity phenotypes. Our study addresses this gap by performing a multi-cancer, transcriptome-integrated analysis of intra-tumour microbial species correlated with body mass index (BMI) and metabolic phenotype. Unlike prior work, we identify associations between microbial abundance and host immune gene expression and pathway activity, revealing both shared and cancer-specific patterns. Furthermore, by stratifying patients into metabolically healthy and unhealthy obese groups, we reveal specific microbial associations, such as protective or inflammatory effects, that were previously undetected.

In this study, we hypothesize that intra-tumour microbes may be a major determinant of clinical course in cancer patients with obesity and investigate the role of BMI-associated microbes in cancer progression, focusing on how specific microbial species correlate with immune-related genes, inflammasome activation and cancer outcomes in patients. By analysing data from The Cancer Genome Atlas (TCGA) for patients with urothelial bladder carcinoma (BLCA), cervical squamous cell carcinoma and endocervical adenocarcinoma (CESC), colon adenocarcinoma (COAD), liver hepatocellular carcinoma (LIHC) and rectum adenocarcinoma (READ), we identified microbes whose abundance was significantly correlated to the metabolic health and obesity of patients. Through various subsequent analyses, we further illuminated multiple possible interactions between the microbiome, obesity and cancer, with findings that suggest that certain microbes associated with obesity may be significantly associated with cancer risk and progression. We hope that these findings can be validated *in vitro* and *in vivo*, opening the door for subsequent research to offer new potential targets for therapeutic strategies in obese patients with cancer.

## Methods

### Data acquisition

Raw TCGA RNA-sequencing data and Level 3 normalized mRNA expression counts for BLCA, CESC, COAD, LIHC and READ tumour samples were retrieved from the GDC Data Portal (https://portal.gdc.cancer.gov/). Clinical patient data were sourced from the Broad GDAC Firehose (https://gdac.broadinstitute.org/), while genomic alteration details for each patient were gathered from the Broad Institute’s TCGA Genome Data Analysis Center report (http://gdac.broadinstitute.org/runs/analyses__latest/reports/).

### Analytical framework and reproducibility

This study is based on reanalysis of publicly available sequencing datasets from TCGA. Such reanalysis constitutes original bioinformatic research, as it integrates microbial abundance, BMI, immune gene expression and clinical outcomes across multiple cancer types in a framework not previously reported. All custom scripts used for these analyses are available from the corresponding author upon reasonable request, ensuring reproducibility of our findings.

### Microbial abundance extraction and analysis

Microbial reads were extracted from the RNA-sequencing data using Pathoscope 2.0, which filtered bacterial sequences through Bowtie2 alignment. Reference bacterial sequences were obtained from the NCBI nucleotide database (https://www.ncbi.nlm.nih.gov/nucleotide/). Pathoscope produced two outputs: ‘best guess’, showing the relative abundance of bacterial species as percentages, and ‘best hit,’ indicating the absolute count of species. To address variability across samples and reduce negative correlation bias, the Aitchison log transformation was applied using the R ‘compositions’ package through RStudio. Known contaminants, including microbes formerly identified as common skin or reagent contaminants, were screened and removed from the dataset [15,16]. While Glassing *et al*. [16] described DNA-based reagent contaminants, these taxa (e.g. *Pseudomonas*, *Ralstonia*, *Cutibacterium*) are widely recognized as universal sequencing artefacts and were therefore used as a broad outline. As our primary guide, we referenced RNA-based contaminant analyses, including Singh *et al.* [15], which characterized microbial contaminants in RNA workflows, and Liu *et al.* [17], which applied the Squeegee algorithm to detect reagent contaminants in human RNA-seq data [17]. By cross-validating removed taxa against both RNA-specific and DNA-based contaminant catalogues, we ensured a conservative and comprehensive filtering strategy appropriate for our dataset.

It should be noted that microbial profiles inferred from RNA-seq represent transcriptional activity rather than total microbial DNA content. Accordingly, our analysis highlights transcriptionally active taxa rather than a complete census of all microbes present. Moreover, species differ substantially in their transcriptional output, which may bias relative abundance estimates. For this reason, our findings are interpreted as associative microbial transcriptional signatures in tumour tissue rather than absolute microbial quantification.

### Differential microbial abundance between cancer and normal patients

To compare microbial abundance between tumour and normal samples, differential analysis was performed, focusing on the percentage of microbial abundance. The Kruskal–Wallis test was employed to determine statistical significance of differential abundance, with a threshold *P*-value of 0.05.

### Correlation of microbial abundance with BMI, survival, clinical data and immune-related genes

Kaplan–Meier survival analysis was conducted, treating microbial presence or absence in tumours as a binary variable. Univariate Cox regression analysis was also used to find microbes significantly associated with survival (*P*<0.05). Lists of immune-related genes were compiled from databases such as ImmPort (http://www.immport.org/), InnateDB (http://www.innatedb.com/) and TANTIGEN (http://cvc.dfci.harvard.edu/tadb/). Correlations between microbial abundance, immune gene expression, BMI and clinical variables were calculated using the Kruskal–Wallis test (*P*<0.05).

### Inflammasome activation analysis

The role of inflammasomes was also assessed in obesity-related cancer progression by determining the correlation between BMI-associated microbes and the expression of inflammasome-related genes such as NFkB and NLRC4. Correlation coefficients were calculated, and significant relationships were determined using the Kruskal–Wallis test (*P*<0.05). The analysis focused on identifying microbial species that either promote or inhibit inflammasome activation, thereby influencing cancer progression in obese patients.

### Functional clustering using ReactomeFIViz

The Cytoscape software, paired with the ReactomeFIViz plugin, was utilized to generate functional gene clusters. Expression data of immune-related genes were input to create a network map, with fold change data modifying the size and outline of the nodes. Gene connections were based on the Reactome database, and the functions of each module were determined by controlling the false discovery rate (FDR<0.05).

### Gene Set Enrichment Analysis

Gene Set Enrichment Analysis (GSEA) was applied to identify biological pathways and molecular signatures related to microbial abundance. Predefined gene sets were sourced from the Molecular Signature Database (https://www.gsea-msigdb.org/gsea/msigdb), and subsequent analysis focused on canonical pathways (C2). The signal-to-noise ratio was used to correlate microbial abundance with the C2 gene set, generating enrichment scores and significance values. The most enriched signatures were identified based on the highest scores and lowest *P*-values (nominal enrichment score >1 and nominal *P*-value<0.05).

## Results

We downloaded the RNA sequencing data for bladder urothelial carcinoma (BLCA), cervical squamous cell carcinoma (CESC), COAD, LIHC and READ from aaaTCGA and ran Pathoscope on the corresponding files to extract microbe abundance data. Intra-tumour microbes have previously been reported to be associated with cancer prognosis and various immune processes. To retain the accuracy of our analyses, we compiled a comprehensive list of immune-associated genes and performed differential analysis on the expression of these genes across our cohorts. Dysregulated genes were then correlated with microbial abundance (Table S1, available in the online Supplementary Material).

### Microbe correlation to cancer patients

**Fig. 1.**
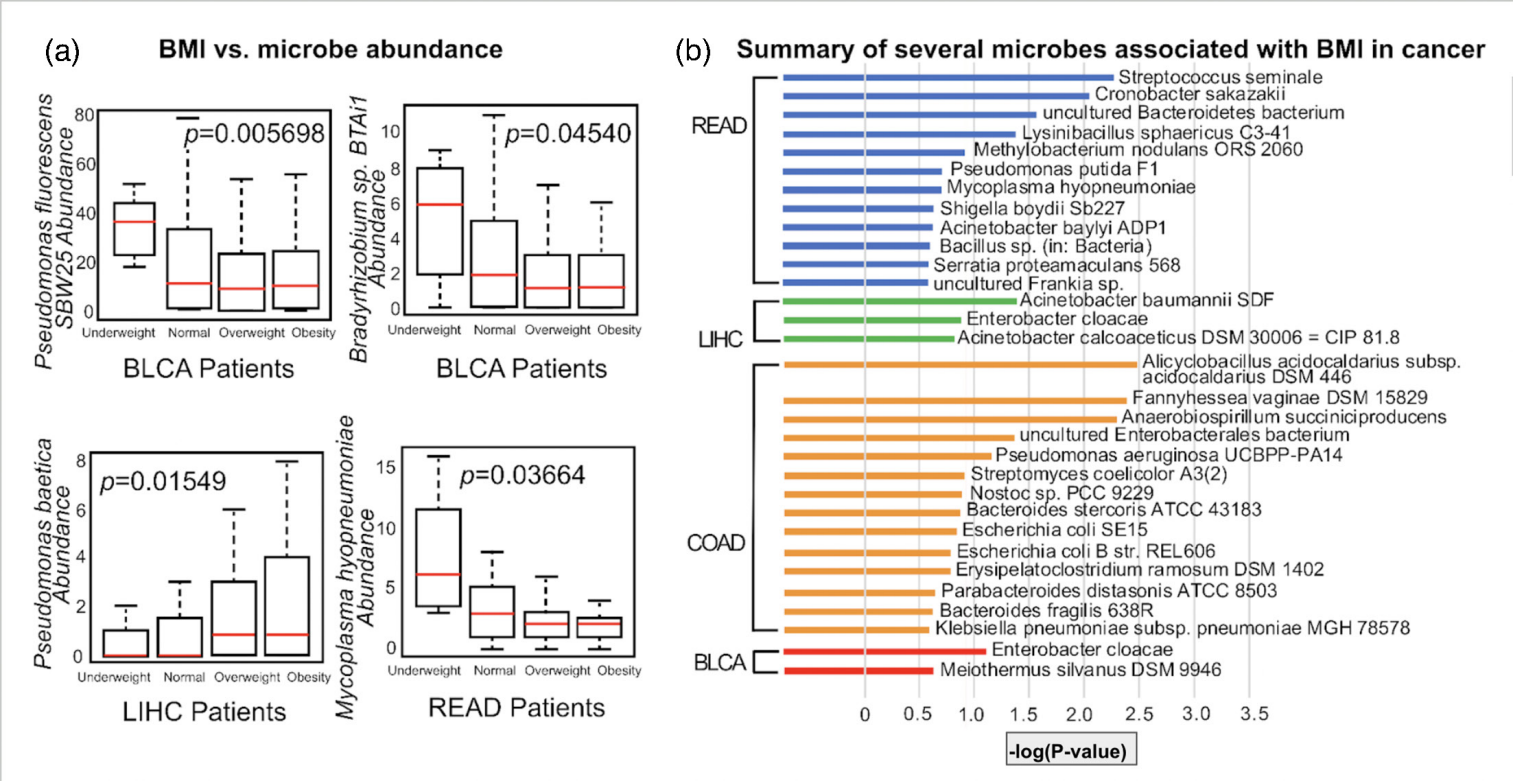
(a) Microbe correlation with BMI bins for cancer samples (Kruskal–Wallis, *P*<0.05). (b) Bar plot of microbes significantly correlated to BMI by -log(*P*-value) in BLCA, COAD, LIHC and READ. (Microbial abundance was inferred from RNA-seq data and represents transcriptional activity, not total microbial DNA content.)

From the results of Pathoscope on our five examined cancer types, we examined intra-tumour microbes through the Kruskal–Wallis test and found several bacterial species associated with high BMI in cancer patients. In bladder cancer, the abundance of *Pseudomonas fluorescens* SBW25 and *Bradyrhizobium* sp. BTAi1 was found to be negatively associated with high BMI, whereas in the liver cohort, *Pseudomonas baetica* abundance was positively associated (Fig. 1a, b).

### Microbe correlation to overall survival and clinical variables in TCGA samples

**Fig. 2.**
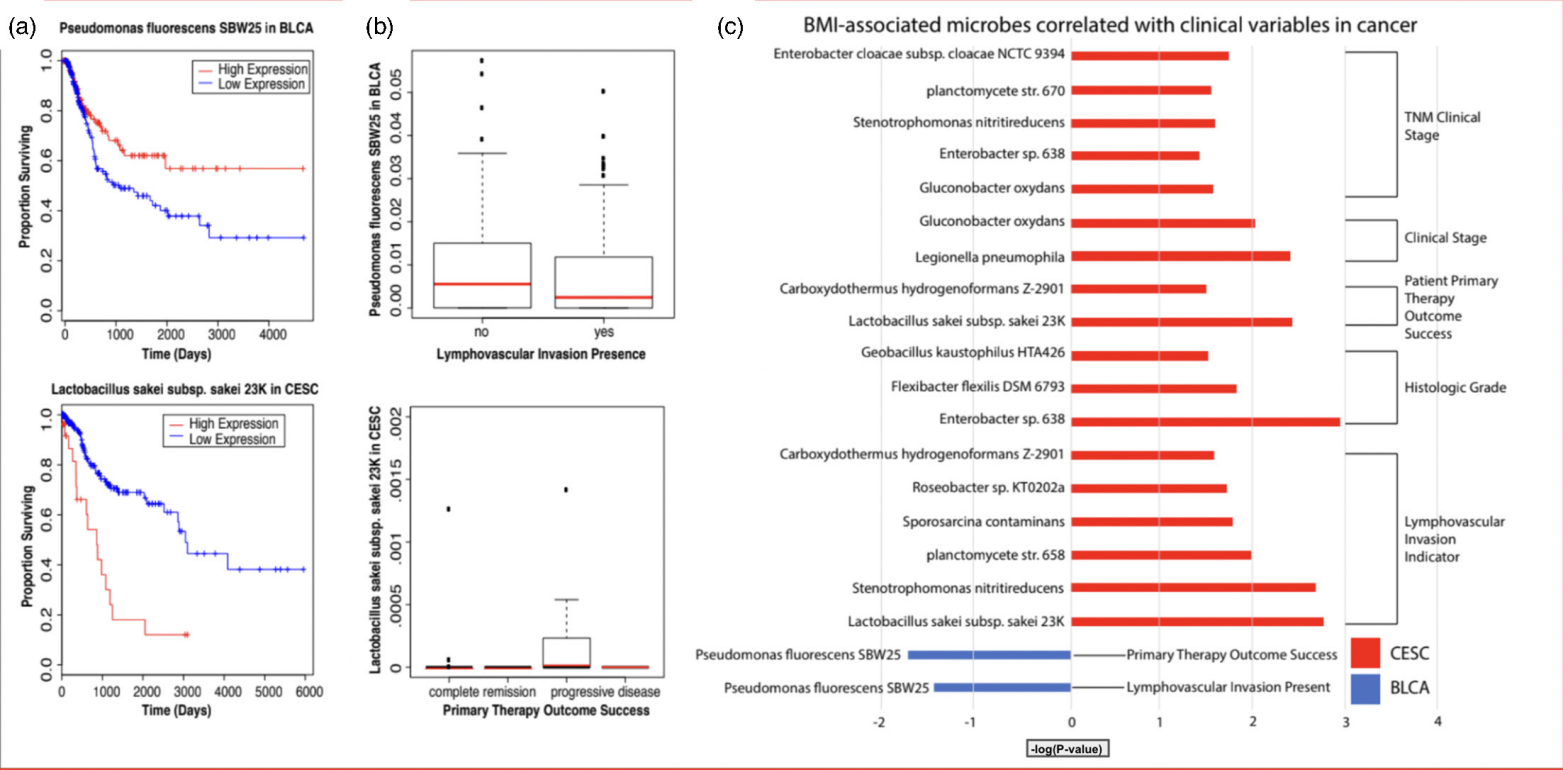
(a) Kaplan–Meier survival plot illustrating correlation between microbe abundance and overall cancer patient survival. (b) Bar plot illustrating differentially abundant BMI-associated microbe species significantly associated with clinical variables in bladder cancer (blue) and cervical cancer (red). Negative correlations in bladder cancer indicate that the microbes have an anti-tumour role, while positive correlations in cervical cancer indicate that the microbes have a pro-tumour role. (Microbial abundance was inferred from RNA-seq data and represents transcriptional activity, not total microbial DNA content.)

Using the Kruskal–Wallis test, we identified the BMI-associated microbes across the patients in our five primary cancer types. In BLCA, the abundance of *P. fluorescens* SBW25 was associated with improved overall survival and a lack of lymphovascular invasion (Fig. 2a, b). Conversely, in CESC, higher levels of *Stenotrophomonas nitritireducens* and *Bacteroides fragilis* YCH46 were linked to lower overall survival and disease progression (Fig. 1a). Microbes that were upregulated in BLCA showed a negative correlation with cancer progression, while those upregulated in CESC were positively associated with several clinical factors, including tumour-node-metastasis stage, histologic grade and lymphovascular invasion (Fig. 2b).

### Correlation of microbial abundance to inflammasome-related genes

**Fig. 3.**
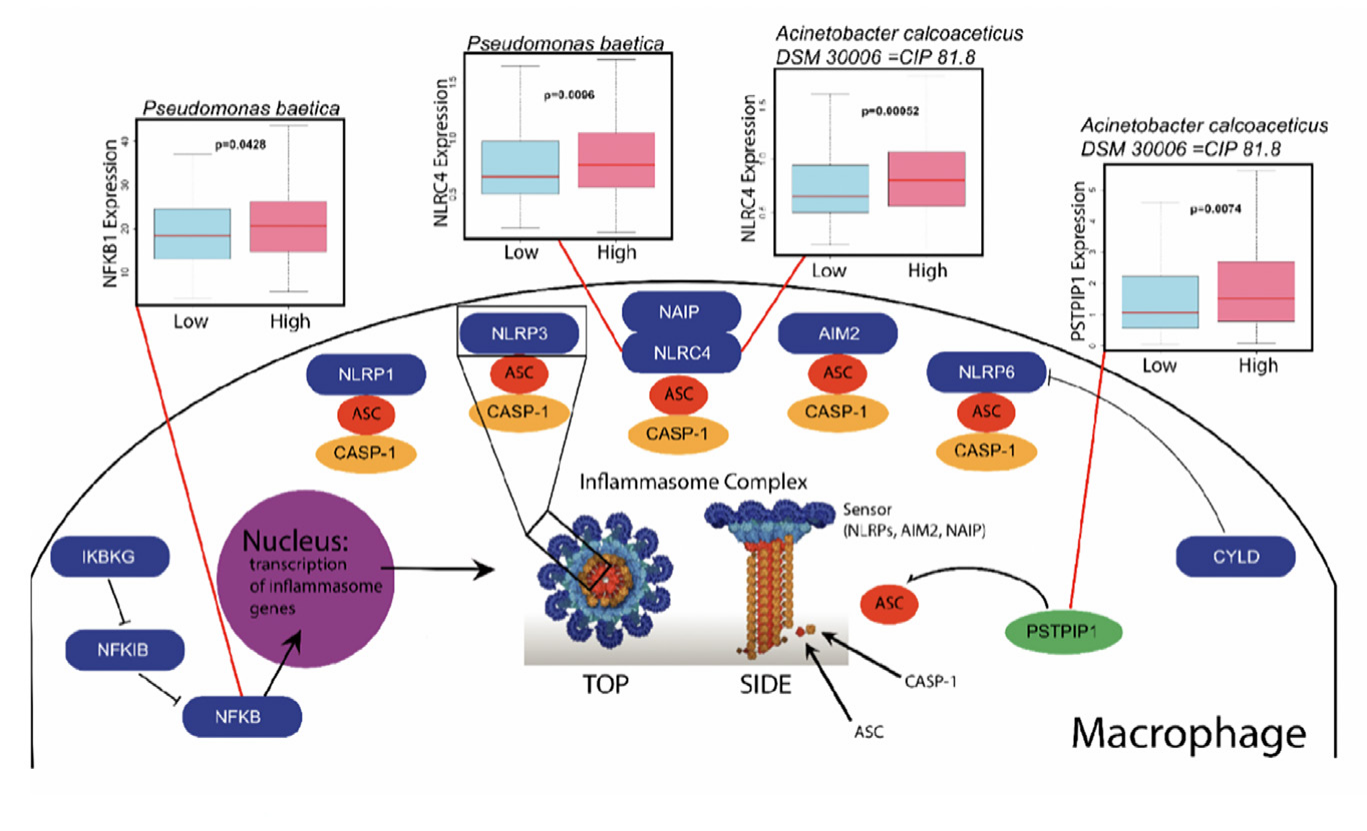
Schematic of obesity-associated microbes correlated with inflammasome-related genes (Kruskal–Wallis, *P*<0.05).

We identified strong correlations between specific microbial species and the expression of inflammasomes – protein complexes primarily found in macrophages that release IL-1β and IL-18 to drive inflammation, a primary driver of cancer development and progression [18]. Notably, in liver cancer, the abundance of *P. baetica* and *Acinetobacter calcoaceticus* showed a significant positive association with inflammasome-related genes, such as *NFkB* and *NLRC4* (Fig. 3). These microbes were also positively correlated with increased BMI, suggesting their potential role in inflammasome activation in obese patients. Interestingly, in metabolically unhealthy obese (MUO) samples, we observed microbes that may play a role in inflammasome deactivation. For instance, *Capnocytophaga canimorsus*, which is positively associated with MUO, was significantly negatively correlated with inflammasome-related gene expression (Fig. 3).

### Microbe correlation to immune-associated genes and immunological pathways

**Fig. 4.**
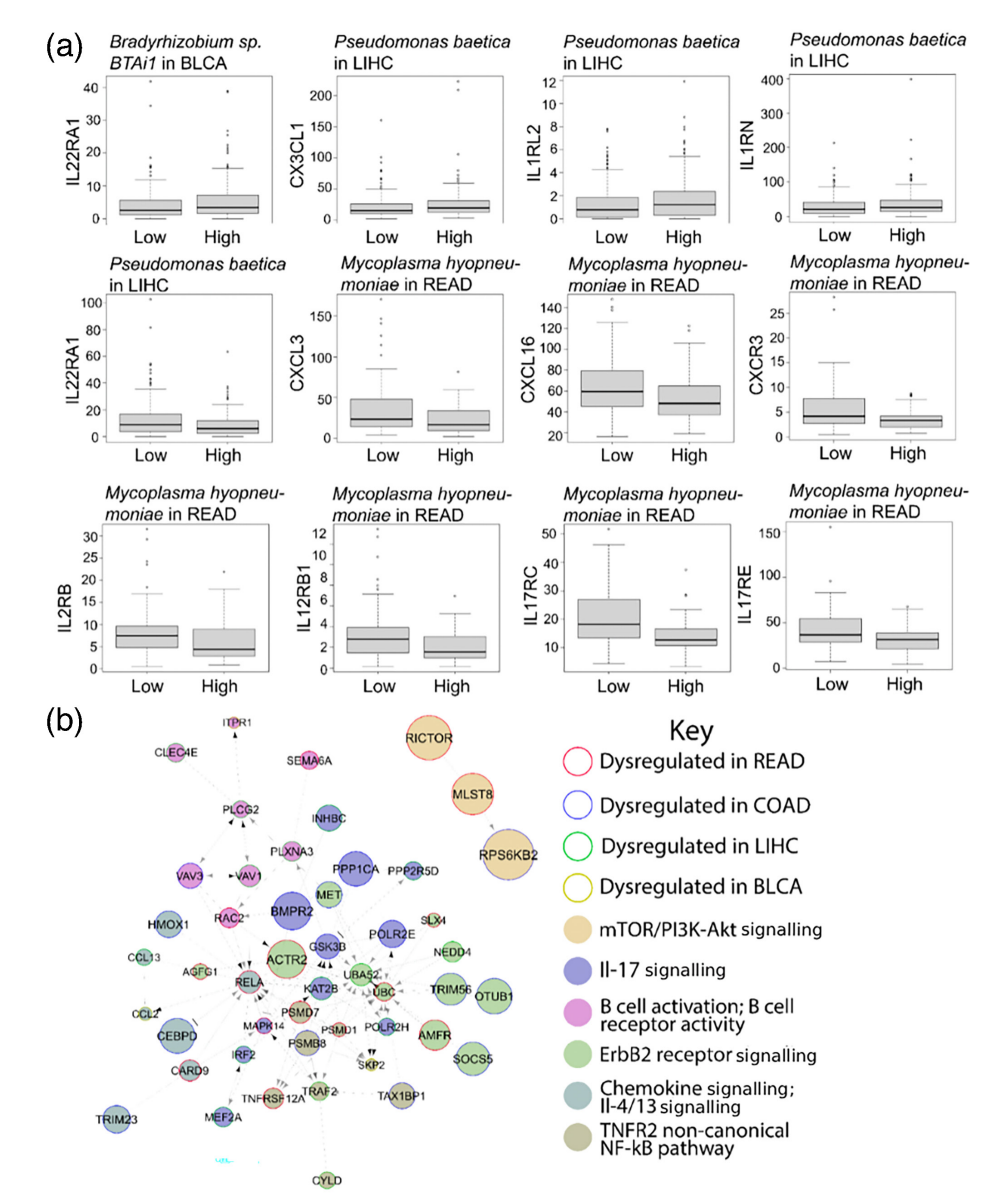
(a) Correlations between BMI-related microbes and immune-associated genes. (b) Pathway map of the top 80 genes correlated with abundance of BMI-associated microbes.

We observed a significant correlation between the abundance of BMI-associated microbes and the expression of hundreds of immune-related genes. In BLCA, *Bradyrhizobium* sp. BTAi1 abundance was linked to elevated expression of *IL22RA1*. In LIHC, *P. baetica* was associated with increased expression of *CX3CL1*, *IL1RL2* and *IL1RN*, while showing an inverse correlation with *IL22RA1* expression. In READ, higher levels of *Mycoplasma hyopneumoniae* were correlated with the downregulation of several key immune genes, including *CXCL3*, *CXCL16*, *CXCR3*, *IL2RB*, *IL12RB1*, *IL17RC* and *IL17RE* (Fig. 4a). Furthermore, the top 80 microbe-correlated immune-associated genes clustered into pathways related to mTOR/PI3K, IL-17 signalling, B cell activity, ErbB2, chemokine signalling and TFR2/NFkB (Fig. 4b).

**Fig. 5.**
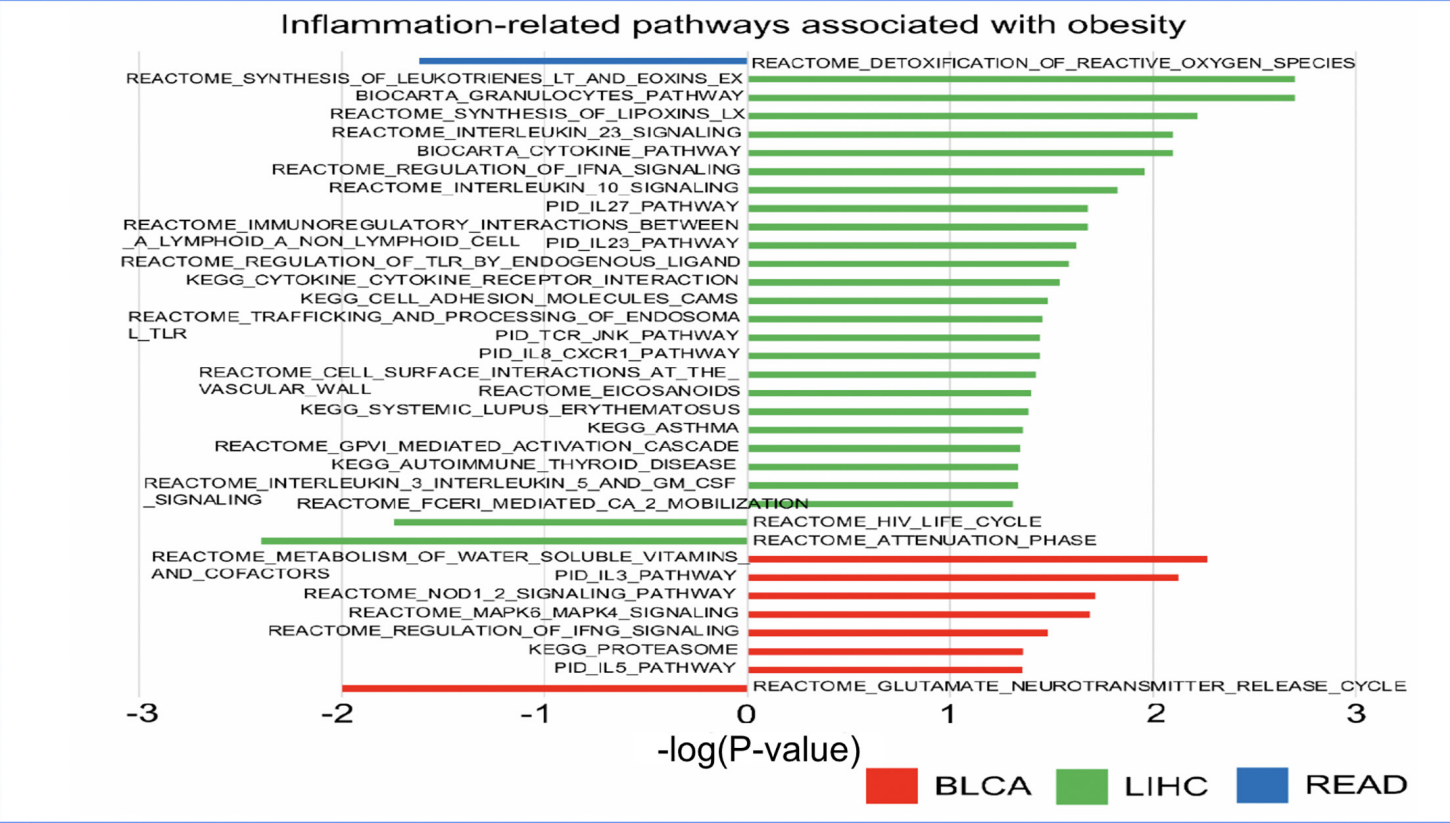
Barplot of inflammation-related pathways correlated with BMI-associated microbe abundance in BLCA, LIHC and READ (Microbial abundance was inferred from RNA-seq data and represents transcriptional activity, not total microbial DNA content).

Subsequently, the BMI-associated microbes also demonstrated significant associations with various metabolic pathways. For instance, *P. fluorescens* SBW25 in BLCA was linked to the synthesis of phosphatidic acid, while *M. hyopneumoniae* in READ was associated with reactive oxygen species detoxification. In LIHC, elevated levels of *Enterobacter cloacae* and *P. baetica* were connected to the upregulation of *MTA3*, mRNA splicing, miRNA biogenesis and Robo receptor signalling. Conversely, lower abundance of these microbes was correlated with the downregulation of methylation, PI3K, PRC2 and IFNA signalling. Notably, in LIHC, high BMI-associated microbial abundance exhibited the strongest positive correlation with several inflammation-related pathways (Fig. 5).

## Discussion

The findings of our study provide important insights into the relationship between BMI-associated microbes, immune response and cancer progression in patients with obesity. Our results show that distinct microbial species are significantly correlated with BMI and are associated with key biological processes involved in both tumour promotion and suppression. For example, in BLCA, *P. fluorescens* SBW25 was associated with improved survival and absence of lymphovascular invasion, which may be consistent with an anti-tumour role for this microbe. In contrast, in CESC, *S. nitritireducens* and *B. fragilis* YCH46 were linked to disease progression and reduced survival, showing an association with a pro-tumour function in this context.

These findings align with existing literature on the microbiome’s role in modulating immune responses in cancer, as previous studies have demonstrated the importance of the gut microbiome in shaping the tumour microenvironment, influencing processes such as inflammation, immune evasion and tissue repair [19,22]. Our study extends this knowledge by identifying associations between BMI-associated microbes and cancer-related pathways, such as those involved in chemokine signalling, mTOR/PI3K signalling and inflammasome activation. These results highlight the complexity of the microbiome’s role in obesity-related cancer progression, where certain microbes may be associated with favourable outcomes, while others were correlated with unfavourable disease characteristics. While previous reviews have postulated the role of gut microbes in modulating cancer immunity and inflammation, our study is unique in directly associating microbial abundance to immune gene expression across multiple cancers and in stratifying these associations by metabolic obesity status. This enables the identification of functionally distinct microbial signatures, including previously uncharacterized pro-inflammatory patterns associated with *P. baetica* in MUO patients.

Our identification of these specific microbes, such as *P. baetica* and *A. calcoaceticus*, which are positively correlated with inflammasome-related genes in LIHC, suggests their association with pro-inflammatory responses. Inflammasome activation has been well-documented as a driver of cancer progression, particularly in obesity, where chronic low-grade inflammation is a hallmark [23,25]. The association between these microbes and inflammasome-related genes supports the hypothesis that microbial dysbiosis in obese patients may exacerbate cancer through heightened inflammatory responses. However, the negative correlation between *C. canimorsus* abundance and inflammasome-related gene expression in MUO samples suggests that certain microbes may conversely play a regulatory role in mitigating inflammation in certain cancer types.

Despite these findings, our study has some limitations. Primarily, the analysis was based on bioinformatic data from TCGA, which, although comprehensive, does not allow for direct mechanistic insights or validation in experimental models. The correlational nature of our study also limits our ability to infer causal relationships between microbes and cancer progression. Future studies should focus on validating these findings *in vitro* and *in vivo* within animal models and clinical samples to determine the precise mechanisms through which BMI-associated microbes may be associated with variations in tumour biology.

In terms of future research directions, there is a need to explore how targeted manipulation of the microbiome – whether through probiotics, prebiotics or microbial metabolites – could be harnessed to prevent or treat cancer in obese patients. Investigating the microbiome’s role across a wider range of cancer types and integrating multi-omic approaches, including metabolomics and proteomics, would provide a more comprehensive understanding of how microbial dysbiosis contributes to the obesity–cancer axis.

## Conclusion

In conclusion, this study highlights the multifaceted role of the microbiome in obesity-driven cancer development, as understanding the interactions between BMI-associated microbes and tumour biology opens new avenues for research and therapeutic strategies. As we continue to explore the microbiome’s impact on cancer, our findings underscore the potential of microbial interventions to alter disease trajectories in patients suffering from both obesity and cancer.

## Supplementary material

10.1099/acmi.0.001055.v4 Uncited Supplementary Material 1.

## Abbreviations

BLCA: urothelial bladder carcinoma
BMI: body mass index
CESC: cervical squamous cell carcinoma and endocervical adenocarcinoma
COAD: colon adenocarcinoma
GSEA: Gene Set Enrichment Analysis
LIHC: liver hepatocellular carcinoma
MUO: metabolically unhealthy obese
READ: rectum adenocarcinoma
TCGA: The Cancer Genome Atlas
